# Association of salivary lactoferrin, *Porphyromoras gingivalis* and stress hormone levels in patients with periodontitis: a pilot study

**DOI:** 10.3389/fimmu.2025.1681095

**Published:** 2025-09-23

**Authors:** Desireé Antequera, Elena Buetas, Sandra García-Esteban, Deborah Romualdi, Laura Carrero, Cristina Municio, Alex Mira, Eva Carro

**Affiliations:** ^1^ Neurobiology of Alzheimer’s DIsease Unit, Chronic Diseases Department-UFIEC, Instituto de Salud Carlos III, Madrid, Spain; ^2^ CIBER for Biomedical Research in Neurodegenerative Diseases (CIBERNED), ISCIII, Madrid, Spain; ^3^ Genomics & Health Department, FISABIO Foundation, Valencia, Spain; ^4^ Programa de Doctorado en Ciencias Biomédicas y Salud Pública, IMIENS, Universidad Nacional de Educación a Distancia (UNED), Madrid, Spain; ^5^ CIBER Center for Epidemiology and Public Health (CIBERESP), ISCIII, Madrid, Spain

**Keywords:** lactoferrin, cortisol, DHEA, porphyromonas gingivalis, periodontal disease, saliva, immune response, oral dysbiosis

## Abstract

**Background:**

Periodontitis is a prevalent inflammatory disease characterized by a dysbiotic oral microbiome, particularly involving *Porphyromonas gingivalis* (*P. gingivalis*) as a key periodontal pathogen. This disorder has also systemic implications, including links to neurodegenerative diseases such as Alzheimer’s disease (AD). Lactoferrin, an iron-binding glycoprotein involved in innate immunity, is found in elevated levels in inflammatory conditions, including periodontitis, and reduced in AD, likely due to hypothalamic-salivary gland axis dysfunction. Additionally, stress-related dysregulation of the hypothalamic-pituitary-adrenal (HPA) axis may contribute to periodontal disease by altering immune responses, notably via elevated salivary cortisol and DHEA levels. The purpose of this study was to evaluate the relationship between salivary immune and stress biomarkers (lactoferrin, cortisol, DHEA) and the abundance of *P. gingivalis* with clinical periodontal parameters.

**Methods:**

A cohort of patients with and without a history of periodontitis was analyzed. Salivary and subgingival biofilm samples were collected to detect *P. gingivalis* levels using 16S rRNA gene sequencing and to quantify salivary biomarker concentrations by ELISA. Common clinical periodontal parameters, including periodontal pocket depth (PPD), clinical attachment level (CAL), bleeding on probing (BoP), and plaque index (PI) were recorded.

**Results:**

Patients with a history of periodontitis showed significantly higher salivary lactoferrin, cortisol and DHEA levels compared to controls, along with increased *P. gingivalis* abundance. Strong correlations were observed between *P. gingivalis* levels and the salivary markers: lactoferrin, cortisol and DHEA. Moreover, lactoferrin, DHEA and cortisol also positively correlated with disease severity, based on the clinical periodontal parameters BoP PPD, CAL, and PI. Similarly, salivary and subgingival *P. gingivalis* positively correlated with BoP, but, specifically, subgingival *P. gingivalis* also correlated with PPD, CAL, and PI.

**Discussion:**

Our findings suggest that elevated lactoferrin, DHEA and cortisol levels reflect both immune-inflammatory and stress-mediated pathways in periodontitis, and its association with the abundance of *P. gingivalis* in saliva and subgingival area. This study supports immune and hormonal dysregulation in periodontal patients, with potential implications in systemic diseases, including such as AD, where lactoferrin levels are seriously altered.

## Introduction

Periodontitis, the most common oral disease in the adult population, is a dysbiotic inflammatory disease. Periodontitis can be classified as aggressive form, with early onset and rapid progression, affecting 8%, or a chronic form, affecting approximately 40% of the adult population (1, 2). In patients with periodontitis, periodontal bacteria, their toxic products, and the locally produced proinflammatory mediators can enter the bloodstream (3), facilitating a potential interaction with other diseases including neurodegenerative diseases (4). The meta-analysis of epidemiological studies showed that subjects with periodontitis have a 1.7 times higher risk of suffering from Alzheimer’s dementia than periodontally healthy individuals.

Periodontitis is a multifactorial disease of polymicrobial origin, however, *Porphyromonas gingivalis* (*P. gingivalis*), a Gram-negative oral anaerobe, is considered a major, keystone periodontal pathogen (5, 6). *P. gingivalis* can locally invade periodontal tissues using a panel of virulence factors that cause deregulation of the innate immune and inflammatory responses (7). Several studies have demonstrated correlation of subgingival and salivary levels of *P. gingivalis* in patients with periodontitis (8, 9). Lactoferrin, an iron-binding glycoprotein, is one of the main antimicrobial proteins identified in saliva, representing the most important factor of natural immunity (10). Lactoferrin has been studied in relation to periodontal disease, and higher concentrations of salivary lactoferrin have been shown in patients with periodontitis (11–13). In such periodontal imbalance, lactoferrin could be released from recruited neutrophils as a potential host defense factor against oral bacteria, as previously suggested (14, 15). Moreover, salivary lactoferrin has been reported as a biomarker of cerebral vulnerability in physiological aging (16) and Alzheimer’s disease (AD) (17–19). Lactoferrin concentration in saliva derives from the secretion of the salivary glands, predominantly from submandibular glands (20). Such secretion depends on the autonomic nervous system control and is connected with the hypothalamus (21). We have reported that AD-related dysfunction of the hypothalamic-salivary gland axis resulted in reduced salivary lactoferrin levels in human and mouse models of AD (22). On the contrary, evidence has shown that lactoferrin concentrations increase in infections and/or inflammatory situations due to the recruitment of neutrophils (15).

Stress or anxiety are also increasingly identified as risk factors that can directly compromise periodontal disease through various biological mechanisms (23). It has been proposed that stress disrupts the balance between pro-inflammatory and anti-inflammatory responses through activation of the hypothalamic-pituitary-adrenal axis (HPA) and the sympathetic nervous system, and finally, this alteration leads to an immunosuppressive action (24). Therefore, stress could be altering the hypothalamic regulation of lactoferrin secretion. On the other hand, stress can lead to the dysregulation of the HPA axis that induces abnormal levels of HPA hormones, including cortisol and dehydroepiandrosterone (DHEA) (25). Cortisol is the most representative stress biomarker, and its higher levels have been associated with oral dysbiosis and decline of immune responses (26). Indeed, cortisol can increase the sensitivity to periodontal pathogens (27). Both salivary cortisol and the other major stress-related biomarker, DHEA, have been associated with periodontal pathology (28, 29). Hence our interest in analyzing the possible relationship between the main stress markers and lactoferrin.

In summary, oral dysbiosis is a central but complex factor in the development and progression of periodontitis, with *P. gingivalis* as the keystone pathogen (30, 31). However, various immunological factors, including impaired HPA regulation, can lead to a loss of microbial homeostasis, associated with alterations in immune and inflammatory responses and pathogen proliferation in the oral cavity (32). In this context, lactoferrin is particularly relevant as it acts as an antioxidant (33), reduces bacterial growth (34, 35), and modulates inflammatory processes (36). The aim of this study was to elucidate the correlation of salivary biomarkers, including lactoferrin and stress markers, and the abundance of *P. gingivalis* with clinical periodontal parameters in a cohort of patients with and without periodontitis.

## Material and methods

### Patients

Forty volunteers were recruited during 2021 and examined by the same odontologist in the Odontology Clinic of Valencia University, Spain. To evaluate the staging and grading of periodontitis, the criteria from Tonetti et al. (37) were followed. Smoking more than 10 cigarettes per day, receiving antibiotic treatment in the past month, using mouth antiseptics in the last 2 weeks or having received periodontal treatment in the last 12 months were exclusion criteria. Twenty donors diagnosed with periodontitis stage III and grade B and twenty individuals with periodontal health (no attachment loss, <10% of bleeding on probing and plaque) were included in the study (**Table 1**). In addition, volunteers filled out a questionnaire about health status, diet, and oral hygiene habits (**Table 2**). All patients agreed with the enrollment and signed the informed consent. The study was approved by the Ethical Committee from the University of Valencia (ref. 1601392).

**Table 1:** Demographic and clinical characteristics of the participants.

		Control (n = 20)	PD (n = 20)	P-value #
Physical characteristic				
Age, mean (SD), y		50.20 (7.66)	52.50 (7.51)	ns
Female/Male		12/8	10/10	ns
BMI (SD)		23.23 (2.80)	26.30 (5.58)	ns
Oral clinical parameters				
PPD, mean (SD), mm		2.32 (0.29)	3.69 (0.53)	<0.0001
CAL, mean (SD), mm		2.38 (0.34)	4.45 (0.91)	<0.0001
BoP, mean (SD), %		4.45 (2.93)	52.40 (17.16)	<0.0001
PI, mean (SD), %		6.85 (2.52)	62.30 (16.80)	<0.0001
Microbiological parameters				
% Porphyromonas (genus), (SD)	Saliva	5.32 (4.14)	9.85 (4.12)	0.002
	Subgingival sulcus	3.13 (4.61)	7.99 (6.12)	0.002
% Porphyromonas gingivalis, (SD)	Saliva	0.71 (2.32)	3.44 (4.32)	0.02
	Subgingival sulcus	0.3 (0.79)	5.44 (5.73)	0.002

PD, Periodontal disease; SD, standard deviation; y, years; n, number; BMI, body mass index; PPD, periodontal pocket depth; CAL, clinical attachment level; BoP, bleeding on probing; PI, plaque index; ns, non-significant. #*p* value indicates statistical difference.

**Table 2:** Health status, diet, and oral hygiene habits of the participants.

		Control (n = 20)	PD (n = 20)	P-value #
Concomitant pathologies				
Hypertension	Yes	3 (0.15)	3 (0.15)	ns
	No	17 (0.85)	17 (0.85)	ns
Cholesterol	Yes	4 (0.2)	3 (0.15)	ns
	No	16 (0.8)	16 (0.8)	ns
Diabetes	Yes	0 (0)	2 (0.1)	ns
	No	20 (1)	18 (0.9)	ns
Food allergys	Yes	2 (0.1)	1 (0.05)	ns
	No	18 (0.9)	19 (0.95)	ns
Intestinal diseases	Yes	4 (0.2)	1 (0.05)	ns
	No	16 (0.8)	19 (0.95)	ns
Routine medication	Yes	9 (0.45)	6 (0.3)	ns
	No	11 (0.55)	14 (0.7)	ns
Antibiotic use in last year	<2 months ago	0 (0)	1 (0.05)	ns
	>2 months ago	5 (0.25)	7 (0.35)	ns
	No	15 (0.75)	12 (0.6)	ns
Halitosis	Yes	0 (0)	5 (0.25)	0.047
	No	19 (0.95)	14 (0.7)	ns
Life style habits				
Alcohol	Never	4 (0.2)	5 (0.25)	0.03
	Hardly ever	1 (0.05)	4 (0.2)	ns
	Sometimes	8 (0.4)	1 (0.05)	ns
	Usually	5 (0.25)	3 (0.15)	ns
	Often	2 (0.1)	7 (0.35)	ns
Tobacco	Yes	4 (0.2)	4 (0.2)	ns
	No	15 (0.75)	12 (0.6)	ns
	Ex	1 (0.05)	4 (0.2)	ns
Physical activity	Never	2 (0.1)	3 (0.15)	ns
	Hardly ever	1 (0.05)	3 (0.15)	ns
	Sometimes	4 (0.2)	4 (0.2)	ns
	Usually	8 (0.4)	5 (0.25)	ns
	Often	5 (0.25)	4 (0.2)	ns
Diet	Mediterranean	17 (0.85)	14 (0.7)	ns
	Vegetarian	2 (0.1)	2 (0.1)	ns
	Vegan	0 (0)	0 (0)	ns
	Others	0 (0)	1 (0.05)	ns
Household location	Rural area	3 (0.15)	5 (0.25)	ns
	Urban area	16 (0.8)	14 (0.7)	ns
Sleep hours	Average a day	6.9	7.3	ns
Water intake	Average a day	1.5	1.61	ns
Bowel movement frequency	<1 a day	4 (0.2)	1 (0.05)	ns
	1 a day	8 (0.4)	12 (0.6)	ns
	2 a day	8 (0.4)	5 (0.25)	ns
	3 or more a day	0 (0)	2 (0.1)	ns
Oral hygienic habits				
Dental brush frequency, n (%)	<1 a day	0 (0)	1 (0.05)	ns
	1 a day	2 (0.1)	5 (0.25)	ns
	2 a day	9 (0.45)	9 (0.45)	ns
	3 or more a day	9 (0.45)	5 (0.25)	ns
Dental floss use, n (%)	Yes	16 (0.8)	5 (0.25)	0.001
	No	4 (0.2)	15 (0.75)	ns
Frequent mothwash use, n (%)	Yes	5 (0.25)	3 (0.15)	ns
	No	15 (0.75)	17 (0.85)	ns

PD, Periodontal disease; ns, non-significant; n, number; #*p* value indicates statistical difference.

**Figure 1:**
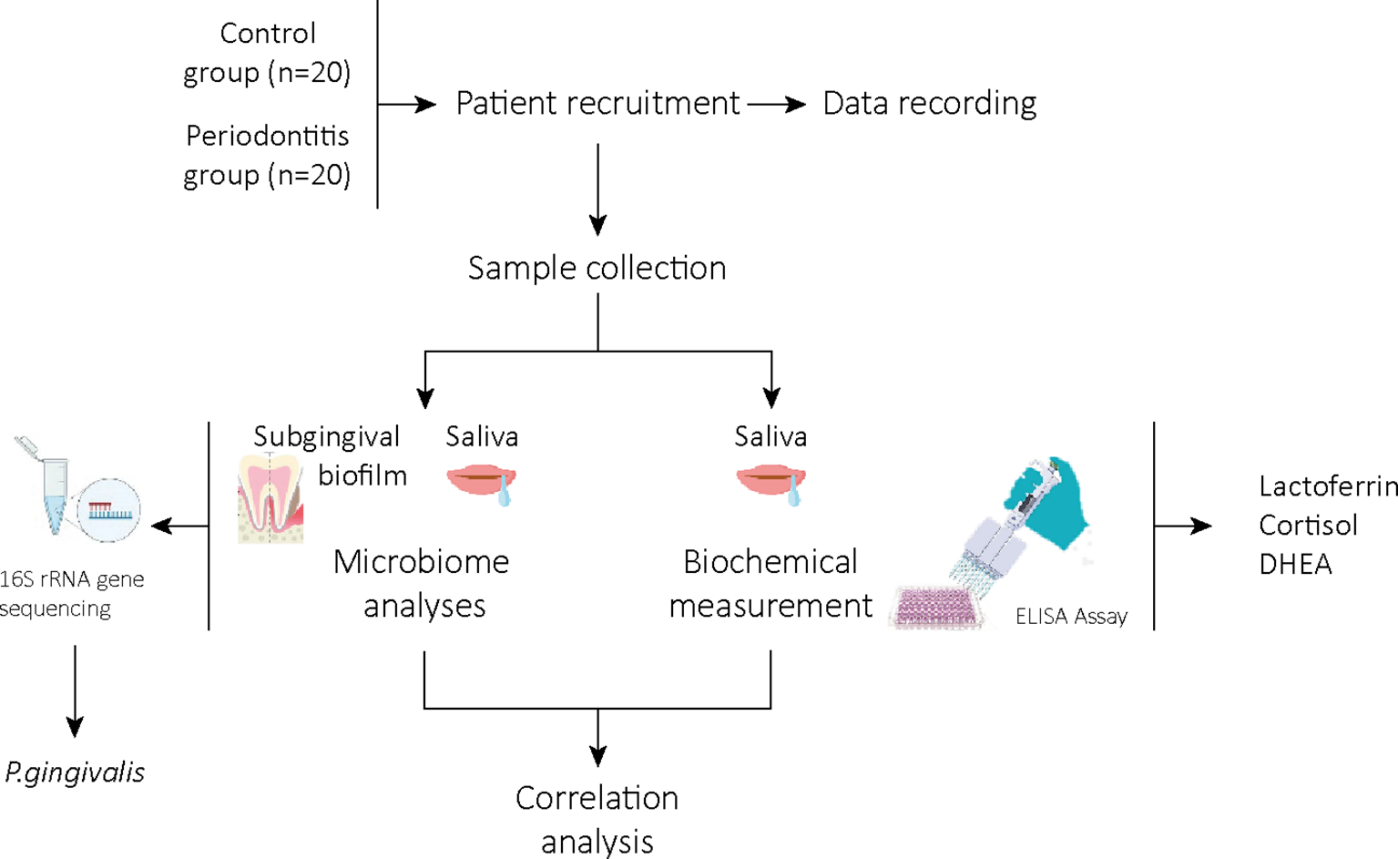
Study work outline. Twenty patients with periodontal and 20 oral healthy individuals were included. Clinical and demographic characteristics were recorded. Saliva and subgingival biofilm samples were taken. Bacterial composition was analyzed by full-length 16S rRNA gene sequencing in both samples. Lactoferrin, cortisol and DHEA were measured in saliva samples using commercial ELISA kits. Correlations were carried out between molecular and clinical parameters.

The study workflow is represented in **Figure 1**.

### Saliva & subgingival biofilm sampling

Up to 2 ml of non-stimulated saliva samples were collected into a sterile plastic tube from each participant in the daytime. Patients were advised to abstain from oral care and eating at least 1 h before saliva collection. After collection, saliva samples were stored at 4°C. Before collecting subgingival samples, supragingival plaque was removed with curettes. Then, four paper points (No. 40) were placed by the odontologist into the periodontal pockets during 1 min and kept in 1 ml of RNAlater (38). On the same day of collection, all sample types arrived at the laboratory and were stored at -80 °C until processing.

### Bacteria detection and quantification

Previous to DNA extraction, subgingival plaque samples were vortexed for 2 min in order to separate bacteria from the paper points. After that, paper points were removed, and the samples centrifuged for 30 min at 13000 rpm. To obtain the bacterial pellet in saliva samples, 250 μl of saliva were centrifuged for 10 min at 13000 rpm. For all sample types, supernatants were removed, and the pellet was resuspended in 100ul of phosphate-buffered saline (PBS). DNA isolation was performed, using the MagNA Pure LC DNA Isolation Kit III for Bacteria and Fungi (Roche Diagnostics, Cat. No. 03 264 785 001) following the manufacturer’s instructions plus an additional enzymatic lysis (39). DNA was quantified using QubitTM 1X dsDNA HS Assay Kit according to the manufacturer’s instructions. Full length 16S rRNA gene sequencing was performed using the Sequel II Sequencing Kit 2.0 (PacBio) on the Sequel II PacBio system (40). Circular consensus sequences were quality checked using PacBio error’s model with DADA2 and annotated using the naive Bayesian classifier against the species train set of Silva v.138.1 database (41). Relative species abundance was calculated as a percentage (n° of reads/total reads*100) for each sample.

### Immunoassays

Levels of salivary lactoferrin, cortisol, and DHEA concentrations were determined from saliva sampling using commercially available specific enzyme-linked immunosorbent assay (ELISA) kits. Levels of human lactoferrin in saliva samples were determined using the human LTF ELISA kit (EH0396, FineTest) according to manufacturer’s instructions. We used a Cortisol Competitive ELISA kit (EIAHCOR, Invitrogen) to measure the human salivary cortisol and the Salivary DHEA ELISA Kit (1-1202, Salimetrics) to measure the human salivary DHEA levels. All samples were measured in duplicate.

### Statistical analysis

Statistical analysis and graphs were performed using GraphPad Prism Software version 8.00 (La Jolla, CA, USA). Descriptive statistics were reported as mean ± standard deviation or numbers with percentages where appropriate. To analyze the distribution of discontinuous data, we used Bonferroni test. Since data were not normally distributed, statistical comparisons between the two independent groups were assessed by Mann-Whitney U test. To determine any relationship between salivary biomarkers and periodontal parameters, and given non-normally distributed data, we used Spearman correlation analysis, and predicted value of these changes were fitted using linear regression models. All p values of less than 0.05 were considered to be statistically significant.

## Results

### Demographics and clinical characteristics

Twenty diseased patients (mean age 52.50 years) and twenty control patients without periodontitis (mean age 50.20 years) were included in this study. No difference between groups according to age, gender and body mass index (BMI) in each group was observed. The demographic data and periodontal clinical parameters of the patients included are provided in **Table 1**. Periodontal registrations included periodontal pocket depth (PPD), clinical attachment level (CAL), bleeding on probing (BoP), and plaque index (PI), clearly exacerbated in subjects suffering from periodontal disease (**Table 1**).

**Figure 2:**
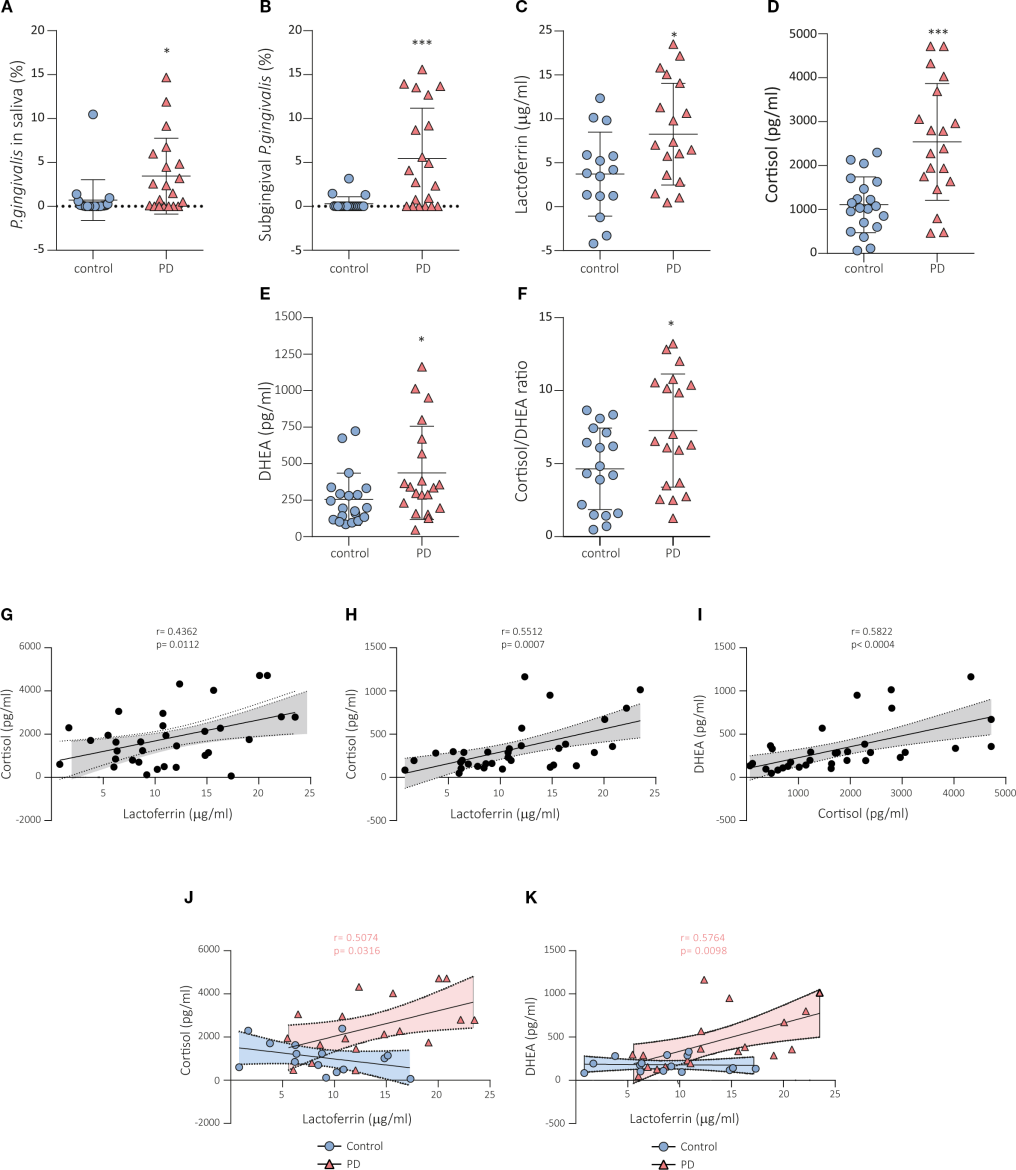
Abundance of *P. gingivalis*, lactoferrin and stress markers in the saliva of subjects with and without periodontal disease. Quantitative analysis revealed significantly higher abundance of *P. gingivalis* in saliva **(A)** and subgingival sulcus **(B)** in patients with periodontitis compared to healthy individuals. Lactoferrin **(C)**, cortisol **(D)**, DHEA **(E)** and cortisol/DHEAR ratio **(F)** levels were significantly higher in saliva from periodontitis cases than in healthy controls. A positive correlation was detected between lactoferrin and cortisol **(G)** and DHEA **(H)** salivary levels in the analysis of the data of the whole cohort. **(I)** Cortisol and DHEA levels were also positively correlated in the whole cohort. However, such correlations between lactoferrin and cortisol **(J)** and DHEA **(K)** salivary levels were only observed in the periodontitis groups and not in the healthy control group. Data are expressed as mean ± SD. Differences between groups were assessed using the Mann-Whitney test. *p < 0.05, ***p < 0.001. PD, periodontal disease; DHEA, dehydroepiandrosterone.

Microbiome differences between oral healthy controls and periodontitis patients were previously described (39). In the present study, we focused on *P. gingivalis* since it is the major etiologic agent which contributes to chronic periodontitis (5, 6). Furthermore, *P. gingivalis* were identified in saliva in 50% of controls and 70% of patients. However, in the subgingival sulcus, *P. gingivalis* was only detected in 5 of the 20 controls, while it was detected in 70% of cases. The relative abundance of *P. gingivalis* was significantly higher in periodontal patients than in healthy individuals in saliva (p < 0.05, **Figure 2A**) and subgingival sulcus (p < 0.001, **Figure 2B**).

### Measurements and correlations of salivary biomarkers

Lactoferrin levels were significantly higher in saliva from periodontitis cases than in healthy controls (p < 0.05, **Figure 2C**). The obtained area under the curve (AUC) was 0.75 (95% CI, 0.58 – 0.91). We also analyzed the total salivary protein concentration, and the ratio of salivary lactoferrin content to total protein. In neither case did we find significant differences between the two study groups. Since stress is associated with periodontitis, stress markers were analyzed by specific immunoassays to confirm their association with periodontal pathology. We found that patients with periodontitis exhibited significantly increased salivary cortisol (p < 0.001, **Figure 2D**), and DHEA (p < 0.05, **Figure 2E**) levels compared to the healthy control group. The cortisol and DHEA AUC were 0.82 (95% CI, 0.68 – 0.96), and 0.70 (95% CI, 0.54 – 0.87), respectively. Moreover, the cortisol/DHEA ratio was also significantly higher in periodontitis patients compared to healthy controls (p < 0.05, **Figure 2F**). Therefore, our finding demonstrated that levels of stress-related factors, cortisol, DHEA, and cortisol/DHEA ratio, and lactoferrin, all of them modulated at hypothalamic level, were higher among patients with periodontitis than in healthy controls.

We found positive correlations between lactoferrin and cortisol (r = 4362; p < 0.05; **Figure 2G**) and DHEA (r = 5512; p < 0.001; **Figure 2H**) levels in the analysis of the data of the whole cohort. Cortisol and DHEA levels were also positively correlated among the 40 participants in the whole cohort (r = 5822; p < 0.001; **Figure 2I**). However, such correlations between lactoferrin and cortisol (r = 5074; p < 0.05; **Figure 2J**) and DHEA (r = 5765; p < 0.01; **Figure 2K**) levels were only observed in the periodontitis groups and not in the healthy control group. The linear regression analyses confirmed these correlations (**Figures 2G-K**).

### Correlations between *P. gingivalis* and salivary biomarkers

**Figure 3:**
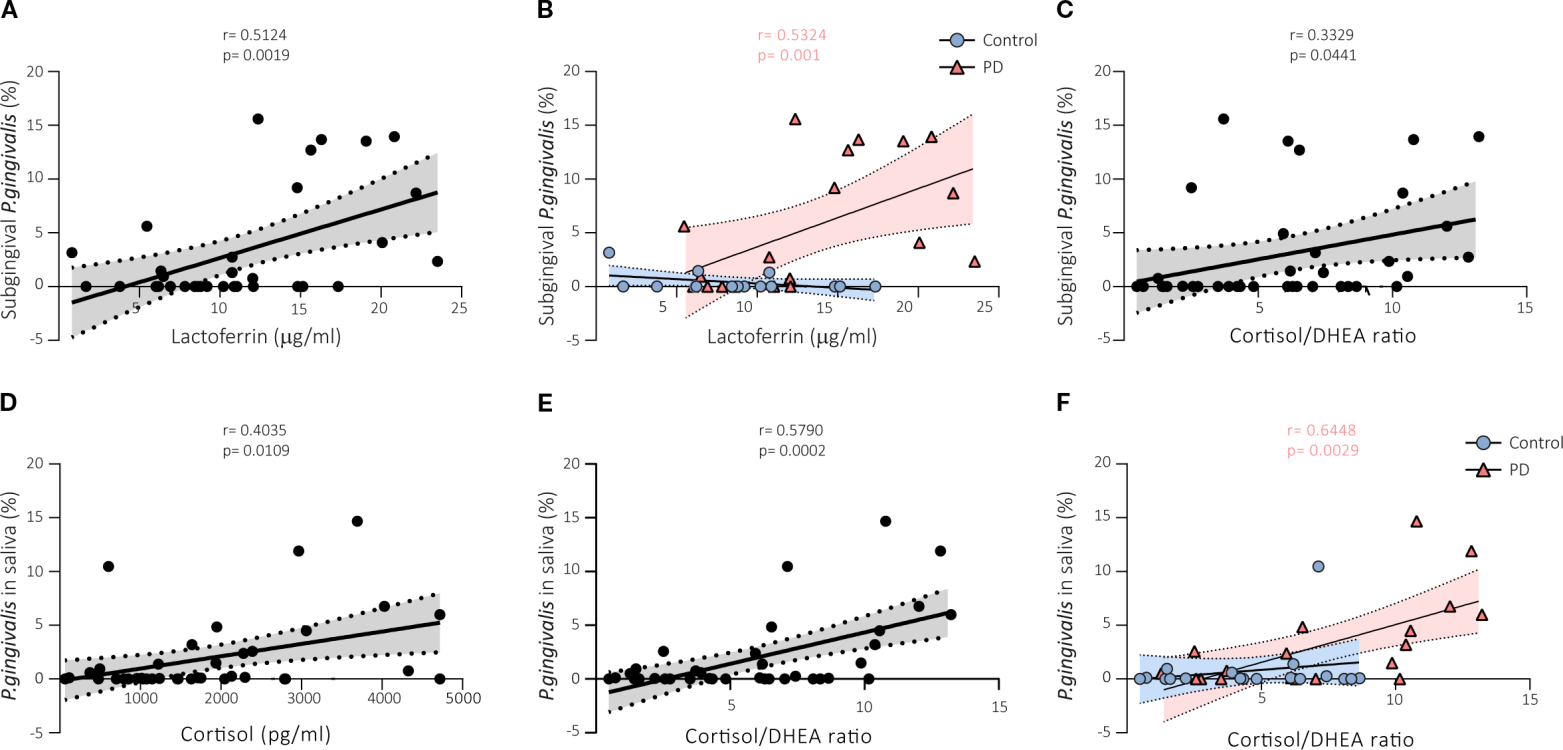
Correlations between *P. gingivalis* and salivary biomarkers markers in the saliva of subjects with and without periodontal disease. *P. gingivalis* in the subgingival area was positively correlated with lactoferrin in the whole cohort **(A)**, and in the periodontitis group **(B)**. **(C)** There was a positive correlation between the abundance of *P. gingivalis* in the subgingival area and the cortisol/DHEA ratio in the whole cohort. *P. gingivalis* in saliva showed a positive significant correlation with cortisol **(D)**, and with the cortisol/DHEA ratio **(E)** in saliva both in the analysis of the data of the whole cohort, and in the periodontitis group **(F)**. PD, periodontal disease; DHEA, dehydroepiandrosterone.

*P. gingivalis* in the subgingival area was positively correlated with lactoferrin both in the analysis of the whole cohort (r = 0.5124, p < 0.0019; **Figure 3A**), and in the periodontitis group (r = 0.5324, p < 0.001; **Figure 3B**) but not in the healthy control group. We also found a positive correlation between the abundance of *P. gingivalis* in the subgingival area and the cortisol/DHEA ratio (r = 0.3329, p < 0.05; **Figure 3C**) in the analysis of the whole cohort but not in the subgroup analyses.

Additionally, *P. gingivalis* in saliva showed a positive significant correlation with cortisol levels in the data analysis of the whole cohort, as shown in the linear regression model (r = 0.4038, p < 0.05; **Figure 3D**). *P. gingivalis* was also positively correlated with the cortisol/DHEA ratio in saliva both in the analysis of the data of the whole cohort (r = 0.5790, p < 0.001; **Figure 3E**), and in the periodontitis groups (r = 0.6448, p < 0.01; **Figure 3F**) but not in the healthy control group.

### Correlations between salivary biomarkers and periodontal parameters

**Figure 4:**
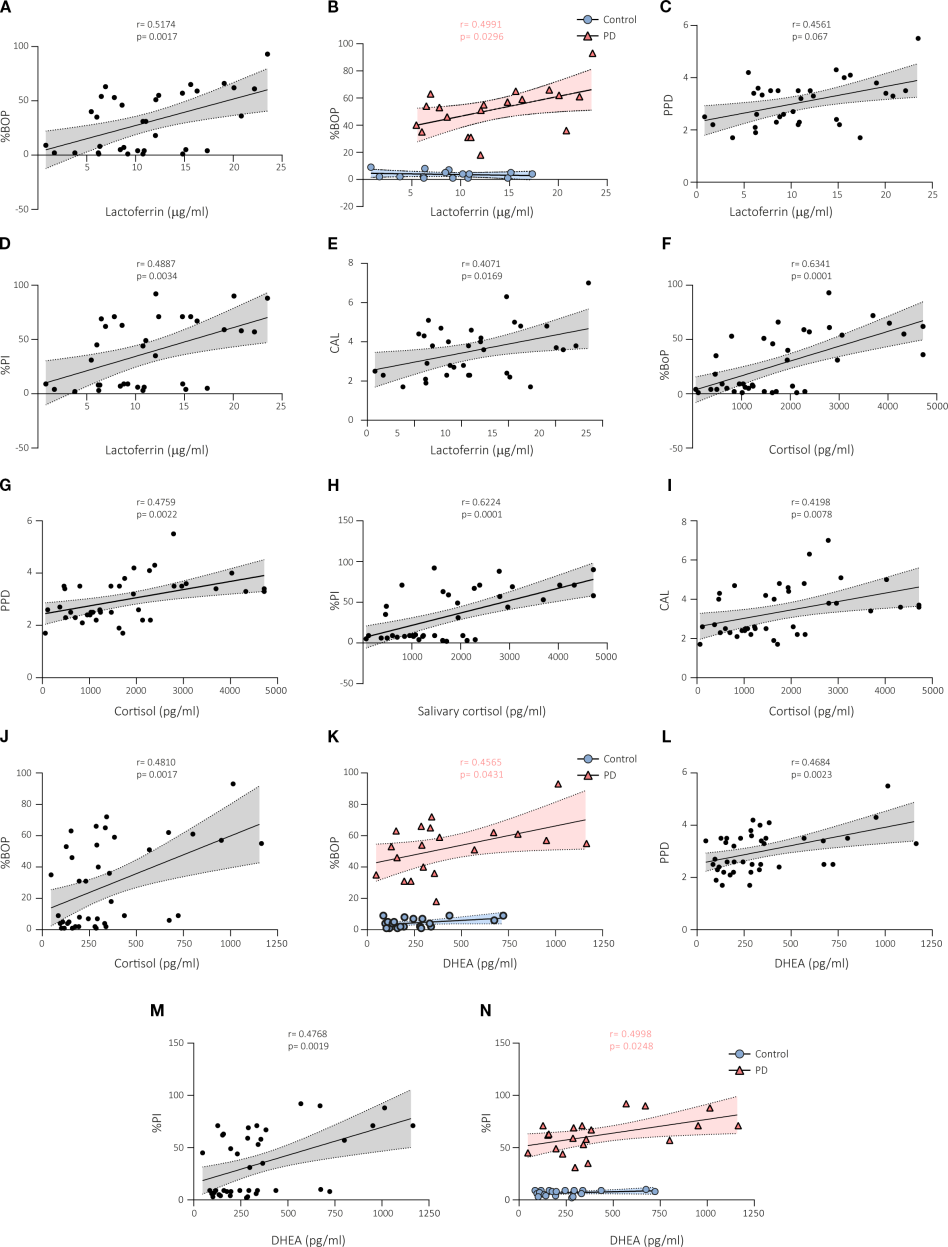
Correlations between salivary biomarkers and periodontal parameters in subjects with and without periodontal disease. A positive correlation was observed between lactoferrin levels and BoP in the whole cohort **(A)** and in the periodontitis group **(B)**. Salivary lactoferrin levels also positively correlated with PPD **(C)**, percentage of plaque or PI **(D)**, and CAL **(E)** in the analysis of the whole cohort but not in the subgroup analyses. Cortisol levels were positively correlated with BoP **(F)**, PPD **(G)**, percentage of plaque **(H)**, and CAL **(I)** in the whole cohort but not in the subgroup analyses. DHEA levels were found to have statistically significant association with BoP in the whole cohort **(J)**, and in the periodontitis group **(K)**. Positive correlation was also found between DHEA and PPD **(L)**, and percentage of plaque **(M)** in the whole cohort. **(N)** Correlation between DHEA and percentage of plaque was detected in the periodontitis group. BoP, bleeding on probing; PPD, periodontal pocket depth; PI, plaque index; CAL, clinical attachment level; PD, periodontal disease; DHEA, dehydroepiandrosterone.

The association of salivary lactoferrin levels with clinical periodontal parameters was evaluated. A positive correlation was observed between lactoferrin levels and BoP in the whole cohort (r = 0.5174, p < 0.01; **Figure 4A**). However, such correlation was only found in the periodontitis group (r = 0.4991, p < 0.05; **Figure 4B**), as expected by the absence of periodontal pockets in the healthy control group (**Figure 4B**). Salivary lactoferrin levels also positively correlated with PPD (r = 0.4561, p < 0.01; **Figure 4C**), percentage of plaque (r = 0.4887, p < 0.01; **Figure 4D**), and CAL (r = 0.4071, p < 0.05; **Figure 4E**) in the analysis of the data of the whole cohort but not in the subgroup analyses.

Similar correlations were observed between salivary cortisol levels and periodontal parameters. We found positive correlations of cortisol levels with BoP (0.6341, p < 0.0001; **Figure 4F**), PPD (r = 0.4759, p < 0.01; **Figure 4G**), percentage of plaque (r = 0.6224, p < 0.0001; **Figure 4H**), and CAL (r = 0.4198, p < 0.01; **Figure 4I**) in the analysis of the data of the whole cohort but not in the subgroup analyses.

Salivary DHEA levels exhibited associations with periodontal parameters similar to those showed above for lactoferrin and cortisol. DHEA levels were not found to have statistically significant association with BoP in the whole cohort (r = 0.4810, p < 0.01; **Figure 4J**), but such correlation was only found in the periodontitis group (r = 0.4565, p < 0.05; **Figure 4K**), and not in the healthy control group, as the lineal regression model indicated (**Figure 4K**). Positive correlation was also found between DHEA and PPD (r = 0.4684, p < 0.01; **Figure 4L**), and percentage of plaque (r = 0.4768, p < 0.01; **Figure 4M**) in the whole cohort. Correlation between DHEA and percentage of plaque was detected in the periodontitis group (r = 0.4998, p < 0.05; **Figure 4N**), and not in the healthy control group, as the lineal regression model revealed (**Figure 4N**).

Altogether, our findings indicate that salivary lactoferrin, cortisol and DHEA can be used to predict the severity of periodontal parameters in periodontitis patients.

### Correlations between abundance of *P. gingivalis* and periodontal parameters

**Figure 5:**
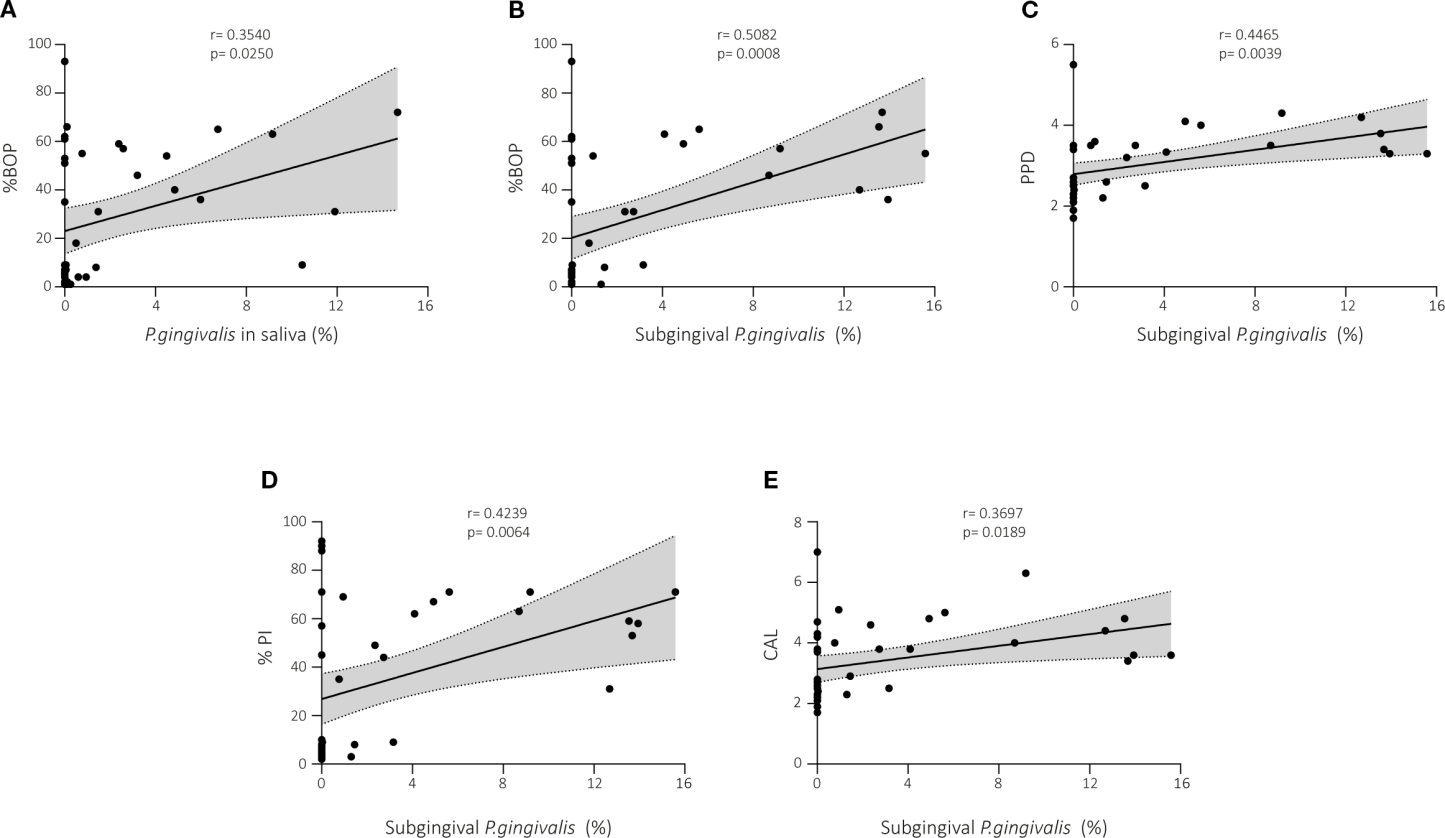
Correlations between abundance of *P. gingivalis* and periodontal parameters in subjects with and without periodontal disease. **(A)** A positive correlation was found between salivary *P. gingivalis* and BoP in the whole cohort. *P. gingivalis* in the subgingival area was positively correlated with BoP **(B)**, PPD **(C)**, percentage of plaque **(D)**, and CAL **(E)** in the whole cohort. BoP, bleeding on probing; PPD, periodontal pocket depth; PI, plaque index; CAL, clinical attachment level.

We assessed whether any correlation exists between clinical periodontal parameters and the abundance of *P. gingivalis* in saliva and the subgingival area (**Figure 5**). **Figure 5A** shows a positive correlation between salivary *P. gingivalis* and BoP in the whole cohort (r = 0.3540, p < 0.05; **Figure 5A**), however no more statistically significant correlations with other periodontal parameters were found.

Regarding the abundance of *P. gingivalis* in the subgingival area, significant associations were observed. BoP (r = 0.5082, p < 0.001; **Figure 5B**), PPD (r = 0.4465, p < 0.01; **Figure 5C**), percentage of plaque (r = 0.4238, p < 0.01; **Figure 5D**), and CAL (r = 0.3697, p < 0.05; **Figure 5E**) correlated with the abundance of *P. gingivalis* in the subgingival area in the analysis of the data of the whole cohort.

## Discussion

The present study identified significant correlations between salivary biomarkers, including lactoferrin and the stress markers cortisol and DHEA, with the abundance of *P. gingivalis* and with clinical periodontal parameters. While previous studies have shown that levels of lactoferrin, cortisol, and DHEA are altered in individuals with periodontitis (12, 28), our study is the first to report a significant interrelationship among these biomarkers. Specifically, we observed that lactoferrin levels correlate with cortisol and DHEA levels in individuals with periodontitis, an association that is not present in healthy subjects. Moreover, our findings show that the abundance of *P. gingivalis* in saliva correlated with lactoferrin, cortisol and cortisol/DHEA ratio. We also observed that periodontal variables were positively correlated with salivary lactoferrin, cortisol, DHEA and *P. gingivalis*. Altogether, these findings suggest a functional interaction between the innate immune response and the neuroendocrine axis in the context of periodontitis, offering a new perspective on the pathophysiological mechanisms of the disease.

The oral microbiome in healthy individuals remains in a constant balance. However, in some cases this balance can become altered, deriving in a pathogenic environment that can contribute to the appearance of oral diseases, such as periodontitis (42). Periodontitis is described as a periodontal dysbiosis resulting from an altered inflammatory environment and a change in microbiota that benefits and amplifies the inflammatory response with a microbiome activity that destroys host tissues through enzymes and inflammatory mediators (43). Among the odontopathogenic bacteria involved in gum diseases, *P. gingivalis*, orchestrates the development of periodontal disease by converting a benign microbial community into a dysbiotic one (30). *P. gingivalis* causes a deregulated immune response due to virulence factors and molecular patterns associated with damage, leading pathogenic dysbiosis and hyperinflammation associated with secretion of proinflammatory cytokines and loss of homeostasis, which leads to connective tissue damage, tooth attachment loss and periodontitis (44, 45).

Lactoferrin plays a crucial role in the innate immune system, exhibiting well-known antimicrobial effects against various bacteria, fungi, and viruses (46). In particular, salivary lactoferrin contributes to maintaining symbiosis between the host and microbiome by regulating the oral microbiota (47). It was documented that lactoferrin inhibits the proteinase activity of *P. gingivalis*, specifically the inhibition of the virulence factor gingipain (48). In our present study lactoferrin levels are increased in saliva samples from patients with periodontitis, accordingly with previous studies (12, 49, 50). It is important to note that lactoferrin is also synthesized by neutrophils that are recruited in inflammatory processes and secreting secondary granules containing lactoferrin (51, 52). And increased neutrophil infiltration has been widely documented in patients with periodontitis (53, 54). Here, we propose that, in addition to salivary glands secretion, increased salivary lactoferrin levels in periodontal condition could be released from recruited neutrophils, based on the strong correlation we found between salivary lactoferrin and subgingival levels of *P. gingivalis*. Lactoferrin is found in saliva but also in gingival crevicular fluid (48). Gingival crevicular fluid is derived primarily from microvascular leakage (55), so neutrophils can enter the gingival crevice (56). Thus, *P. gingivalis*, that resides predominantly in subgingival biofilms (57), could facilitate the entry of neutrophils from the blood and therefore, the salivary levels of neutrophil-derived lactoferrin.

In periodontal disease it has been reported that stress-markers, including cortisol and DHEA, compromise immune response, affecting oral microbiome (23, 25, 58). Since the main source of salivary lactoferrin is the salivary glands, which are closely linked to the HPA control (21, 59), we suggest that stress-mediated risen cortisol and DHEA expression can indirectly induce greater secretion of lactoferrin into saliva mediated by salivary gland upregulation. Our findings also revealed a significant correlation between salivary levels of cortisol and *P. gingivalis*, according to a previous study (60). Cortisol has been suggested to modify composition of subgingival biofilms. Particularly, cortisol was able to significantly increase *P. gingivalis* growth in culture (27). Altogether, we suggest that the high levels of lactoferrin found in our present study can be explained by increased secretion from both molecular mechanisms: HPA-mediated salivary gland activation and infiltrated neutrophils into the gingival crevicular fluid, probably as a compensatory mechanism to recover oral homeostasis. However, since neither total protein levels protein levels nor the ratio lactoferrin/total protein varied significantly between the periodontal disease group and the control group, we propose that the elevated salivary lactoferrin levels were not due to increased production by the salivary glands but to the release from infiltrated neutrophils. In any case, further studies will be needed to demonstrate this hypothesis.

In our study, salivary lactoferrin levels were positively correlated with periodontal variables, including BoP PPD, CAL, and PI. These results were consistent with previously reported findings although only the correlation of lactoferrin with BoP and PPD was described (12). Our results extend and demonstrate that salivary lactoferrin positively correlated with CAL, and PI. Notably, salivary lactoferrin was only correlated with bleeding in the periodontal disease group, which would support our hypothesis that high levels of lactoferrin found in saliva from periodontal disease patients can be explained by neutrophil infiltration due to microvascular leakage. Our study also confirmed that salivary cortisol and DHEA levels were correlated with BoP PPD, CAL, and PI, confirming but also expanding additional findings. Meanwhile previous studies have reported correlations between salivary cortisol and periodontal clinical parameters (29, 61), no data was found related to salivary DHEA correlations.

Additionally, we found that salivary and subgingival abundance of *P. gingivalis* was positively correlated with BoP, but, specifically, subgingival *P. gingivalis* also correlated with PPD, CAL, and PI. Since *P. gingivalis* colonizes predominantly subgingival biofilms as periodontal disease risk factor (57, 62, 63), higher subgingival *P. gingivalis* may drive the tissue destruction in periodontal disease patients. The correlation between periodontal parameters and *P. gingivalis* abundance has been described in individuals hospitalized in the intensive care unit, who underwent a complete periodontal examination and microbiological sampling, and showing positive correlation with BoP and PPD (64).

This study had several limitations. First, this is a case-control study with its analysis restricted to a specific age range, however, age and sex are confounding variables that could influence. Second, the sample size in our study (n = 20 per group) may limit the analysis of correlations. Thus, further studies will be needed that apply stratification that allows the study population to be divided into age groups, and to evaluate neuropsychological variables and other clinical confounders. Additionally, further investigations are required to clarify the source of increased salivary lactoferrin levels in periodontal condition, including the role of infiltrated neutrophils from microvascular leakage and HPA-mediated salivary gland regulation.

In conclusion, our data indicates that salivary lactoferrin appears to be associated with stress-related hormones cortisol and DHEA, and abundance of *P. gingivalis* in periodontal disease patients. Furthermore, we found intercorrelations between these biochemical markers and clinical periodontal parameters highlighting the multifactorial feature of periodontal disease associated with oral dysbiosis and the progressive damage of the tooth-supporting tissue. This provides important information about the influence of both local and systemic factors and supports that periodontitis influences not only oral bacterial composition, but also salivary lactoferrin and stress-related hormones with potential effects for other diseases, such as AD, where lactoferrin levels are seriously altered.

## Data Availability

The original contributions presented in the study are publicly available. Or All data are available in the main text. The original sequencing data was obtained from Buetas et al. 2024 available in the SRA repository with the accession number PRJNA933120.
